# Time-dependent alteration in the chemoreflex post-acute lung injury

**DOI:** 10.3389/fphys.2022.1009607

**Published:** 2022-10-20

**Authors:** Kajal Kamra, Nikolay Karpuk, Ryan Adam, Irving H. Zucker, Harold D. Schultz, Han-Jun Wang

**Affiliations:** ^1^ Department of Cellular and Integrative Physiology, University of Nebraska Medical Center, Omaha, NE, United States; ^2^ Department of Anesthesiology, University of Nebraska Medical Center, Omaha, NE, United States

**Keywords:** acute lung injury, acute respiratory distress syndrome, bleomycin, chemoreceptors, chemoreflex, carotid bodies

## Abstract

Acute lung injury (ALI) induces inflammation that disrupts the normal alveolar-capillary endothelial barrier which impairs gas exchange to induce hypoxemia that reflexively increases respiration. The neural mechanisms underlying the respiratory dysfunction during ALI are not fully understood. The purpose of this study was to investigate the role of the chemoreflex in mediating abnormal ventilation during acute (early) and recovery (late) stages of ALI. We hypothesized that the increase in respiratory rate (fR) during post-ALI is mediated by a sensitized chemoreflex. ALI was induced in male Sprague-Dawley rats using a single intra-tracheal injection of bleomycin (Bleo: low-dose = 1.25 mg/Kg or high-dose = 2.5 mg/Kg) (day 1) and respiratory variables- fR, V_t_ (Tidal Volume), and V_E_ (Minute Ventilation) in response to 10% hypoxia (10% O_2_, 0% CO_2_) and 5% hypercapnia/21% normoxia (21% O_2_, 5% CO_2_) were measured weekly from W0-W4 using whole-body plethysmography (WBP). Our data indicate sensitization (∆f_R_ = 93 ± 31 bpm, *p* < 0.0001) of the chemoreflex at W1 post-ALI in response to hypoxic/hypercapnic gas challenge in the low-dose bleo (moderate ALI) group and a blunted chemoreflex (∆f_R_ = −0.97 ± 42 bpm, *p* < 0.0001) at W1 post-ALI in the high-dose bleo (severe ALI) group. During recovery from ALI, at W3-W4, both low-dose and high-dose groups exhibited a sensitized chemoreflex in response to hypoxia and normoxic-hypercapnia. We then hypothesized that the blunted chemoreflex at W1 post-ALI in the high-dose bleo group could be due to near maximal tonic activation of chemoreceptors, called the “ceiling effect”. To test this possibility, 90% hyperoxia (90% O_2_, 0% CO_2_) was given to bleo treated rats to inhibit the chemoreflex. Our results showed no changes in f_R_, suggesting absence of the tonic chemoreflex activation in response to hypoxia at W1 post-ALI. These data suggest that during the acute stage of moderate (low-dose bleo) and severe (high-dose bleo) ALI, chemoreflex activity trends to be slightly sensitized and blunted, respectively while it becomes significantly sensitized during the recovery stage. Future studies are required to examine the molecular/cellular mechanisms underlying the time-course changes in chemoreflex sensitivity post-ALI.

## Introduction

Acute lung injury (ALI) and its clinical correlate, the acute respiratory distress syndrome (ARDS), results due to disruption of the normal capillary endothelial barrier and invokes perturbations of ventilatory control (Young et al., 2019). Acute respiratory failure affects approximately 200,000 new cases each year in the US alone and accounts for 10% of ICU admissions with a high mortality and morbidity (Mowery et al., 2020). Current treatment therapies are focused on resolution of the lung disorder by fluid management, prone positioning, pharmacological interventions, and mechanical ventilation. ALI/ARDS causes marked diffuse alveolar damage, endothelial cell damage and pulmonary interstitial and alveolar edema that results in increased intrapulmonary shunt and dead space as well as atelectasis leading to a decreased functional lung size (Bernard et al., 1994; Ghio et al., 2001; Spinelli et al., 2020). This causes an impairment in gas exchange inducing hypoxemia, a hypoxic ventilatory response (HVR) and a reflexive increase in respiratory rate (f_R_) (Bernard et al., 1994; Jacono et al., 2006; Spinelli et al., 2020). The neural mechanism that drives this increase in f_R_ during ALI is not fully understood.

Peripheral chemoreceptors in the carotid bodies (CBs) located at the bifurcation of the common carotid artery, are the first responders to hypoxia. A decrease in arterial pO_2_ causes the glomus cells (type 1 cells) of the CBs to depolarize, increase intracellular calcium levels and closure of potassium channels to cause neurotransmitter release. The chemosensory afferent input from glomus cells then travels to the respiratory network in the brain stem through the carotid sinus nerve (CSN) which projects to the nucleus solitarius tractus (NTS) and respiratory motoneurons to induce a hypoxic ventilatory response (Sun et al., 1999; Prabhakar, 2000). The HVR in the early stage of ALI has been examined (Jacono et al., 2006; Huxtable et al., 2011) but little is known about the exact time-dependent changes in chemoreflex sensitivity in acute ALI and during the recovery of ALI. The specific objective of our study was to look at the time dependent respiratory changes in chemoreflex function in response to hypoxic and hypercapnic stimuli before ALI (Week 0; W0) and weekly for up to 4 weeks (W1-W4) post-ALI.

## Methods

### Ethical approval

Animals were housed in a temperature-controlled environment (22°C–25°C) with a 12 h light–dark cycle and *ad libitum* access to food and water, in accordance with standards set by the National Institutes of Health Guidelines for the Care and Use of Laboratory Animals. All experimental protocols were approved by the Institutional Animal Care and Use Committee (IACUC) of the University of Nebraska Medical Center (protocol ID no. 17-006-03 FC).

### Animals

**FIGURE 1:**
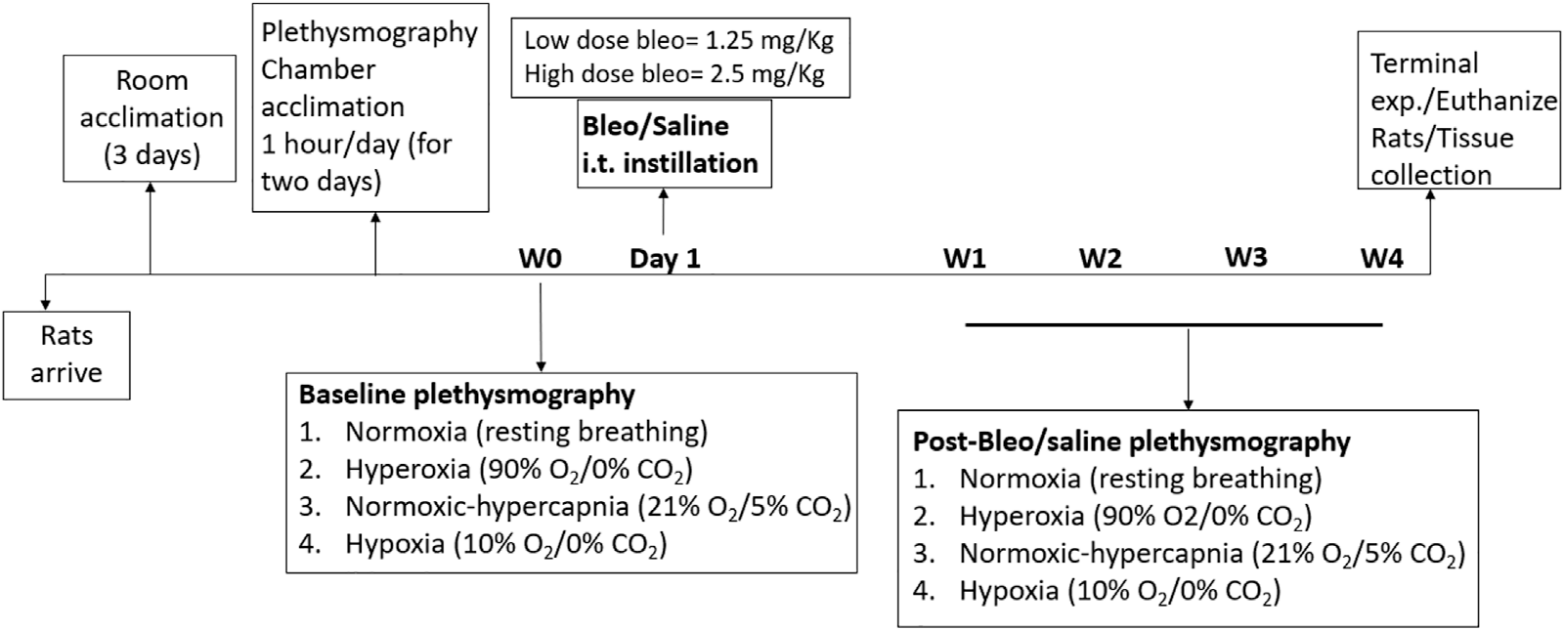
Timeline showing experimental design.

Thirty-three adult male Sprague-Dawley rats (2–3 months old) were used for these experiments. Animals were housed on-site in a controlled temperature environment (22°C–25°C) with a 12-h light-dark cycle and *ad libitum* access to food and water and were allowed to acclimate for 3 days to their new environment prior to the experiment. A small number of rats (1 high-dose bleo rat) died during experimentation after W2 post-bleo administration stemming from severe lung injury. All animal experimentation (collection of ventilatory parameters during rest and during hypoxic/hypercapnic gas exposure) was performed during the day (9.00–1600 h). Delivery of bleo (or saline) was performed within our animal housing center. At the end of the experimental protocol, all animals were humanely euthanized with an overdose of pentobarbital sodium (150 mg/kg, IV). Euthanasia was confirmed by removal of vital organs and lung tissue was collected for further analysis. An experimental timeline is shown in Figure 1.

### Drugs and chemicals

Bleomycin sulphate (bleo) was purchased from Enzo Life Sciences (New York, United States). Bleo was dissolved in saline for intra-tracheal administration. This procedure was performed within the animal housing center.

### Rat model of lung injury

Rats were randomized into three experimental groups and lung parameters were measured at five time points- before (W0) and post-instillation (W1, 2, 3 and 4) as follows: sham rats (*n* = 14), low-dose bleo-treated rats (*n* = 10) and high-dose bleo-treated rats (*n* = 9). Bleo (2.5 mg/kg (high-dose) and 1.25 mg/kg (low-dose), ∼0.15 ml) was instilled on day 1 intra-tracheally under 2%–3% isoflurane anesthesia. Sham control animals underwent intra-tracheal instillation of saline (∼0.15 ml).

### Breathing and ventilatory chemoreflex function at rest

Unrestrained whole-body plethysmography was utilized to measure ventilatory parameters- respiratory rate (f_R_), tidal volume (V_t_) and minute ventilation (V_E_) in conscious rats by using signals from differential-pressure transducer (DLP 2.5, Harvard Apparatus), amplified and connected to PC *via* acquisition system PowerLab 35 Series managed by LabChart (v8.1.5) software (ADInstruments, Colorado, United States). Rats were acclimated to the plethysmograph chamber for 1 h each for two consecutive days prior to recordings. Respiratory parameters were not recorded during the acclimatization sessions. The plethysmograph chambers used for this study were custom-made (Midwest Plastics Inc., Nebraska, United States) and were 10, 10.5 and 20 cm in height, width, and length, respectively. The volume channel (i.e., flow integration) was calibrated by pushing 5 ml of air using a syringe before the start of the recording. During recordings, a constant flow of gas at 3 L/min was maintained to avoid an increase in humidity, temperature and CO_2_ levels using a manually operated flow meter (Precision Medical, Northampton, PA, United States). Body weight (in grams) of rats was recorded prior to each experiment. In the resting state rats were exposed to normoxia (21% O_2_, 0% CO_2_) for baseline measurements followed by three different gas challenges- hyperoxia (90% O_2_, 0% CO_2_), hypoxia (10% O_2_, 0% CO_2_) and normoxic hypercapnia (21% O_2_, 5% CO_2_) balanced by N_2_. The order of gas challenge was randomized and was maintained for 5 min. The last one-minute-long segment without any artifacts was used for analysis. A normoxic exposure of a minimum of 10 min or more was used in between challenges. All resting ventilatory parameters considered for analysis were recorded when the rats were awake and stationary (no activity-related events recorded in LabChart8 raw data file). V_E_ was calculated as the product of fR and V_t_. V_t_ and V_E_ were normalized to bodyweight.

### Statistical analysis

Data analysis in text, tables and figures are presented as mean ± SD. Statistical evaluation was analyzed using GraphPad Prism (GraphPad Software, San Diego, CA. Version 8). Comparisons between conditions (gas challenges) and for comparisons between groups (Sham, low-dose bleo and, high-dose bleo) repeated-measures two-way ANOVA with bonferroni corrections for multiple comparisons were used with *p* < 0.05 being statistically significant. For tables, the comparison of body weight and resting fR at each timepoint post-drug (bleo/saline) treatment with W0 (pre-treatment) within each experimental group was done using one-way ANOVA with bonferroni multiple comparison test with *p* < 0.05 being statistically significant.

## Results

### Effects of saline and bleomycin on body weight in rats

Body weights were measured weekly in all rats pre- (W0) and post-saline/bleo instillation (W1, 2, 3 and 4). At W1-post-bleo instillation, low-dose bleo-treated rats (∆ body weight at W1 post-ALI = 6.6 ± 50 g) showed no significant changes but high-dose bleo-treated rats (∆ body weight at W1 post-ALI = −19 ± 20 g) showed a reduction in body weight that was not statistically significant when compared to change in body weight in sham rats at W1-post-saline/bleo instillation (∆ body weight from W0 to W1 post-ALI = 69 ± 20 g) (Table 1). Sham and low-dose bleo-treated rats continued to gain weight each week throughout the experimental timeline (Table 1; Figure 1). High-dose bleo-treated rats gained weight by the end of W3 and W4 (Table 1). One high dose bleo-treated rat died at W2.

**TABLE 1:** Mean body weight and mean change in body weight (in grams) for sham, low-dose bleo and high-dose bleo rats.

	Mean body weight (grams)					Mean Δ body weight (grams)			
	W0	W1	W2	W3	W4	ΔW1	ΔW2	ΔW3	ΔW4
Sham (*n* = 14)	337 ± 41	405 ± 42***	443 ± 45****	472 ± 45****	485 ± 48****	69 ± 20	107 ± 27	136 ± 36	148 ± 35
Low-dose bleo (*n* = 10)	465 ± 114	472 ± 80	488 ± 66	506 ± 62	522 ± 55	6.6 ± 50	23 ± 60	41 ± 61	57 ± 65
High-dose bleo (*n* = 9)	301 ± 66	283 ± 71	313 ± 93	386 ± 65	412 ± 55ǂ	-19 ± 20	11 ± 56	77 ± 23	103 ± 21

Values are mean ± SD; One-way ANOVA with bonferroni multiple comparison test; bleo indicates bleomycin. ***p = 0.0004 (Sham-W0 vs. W1), ****p < 0.0001 (Sham- W0 vs. W2, W3 and W4), ǂ*p* = 0.0117 (High-dose bleo- W0 vs. W4).

### Bleomycin caused an increase in resting f_R_

**FIGURE 2:**
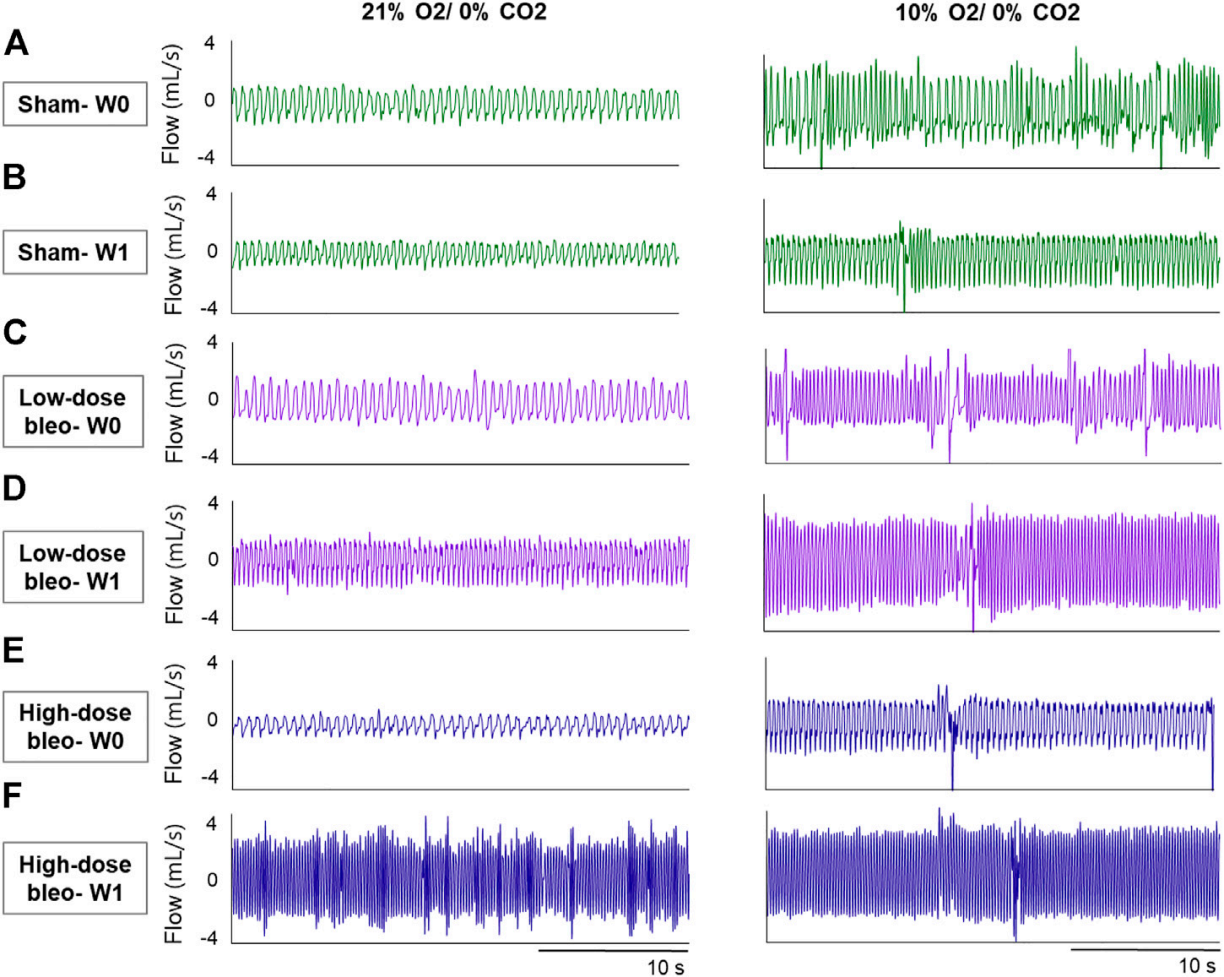
Representative recordings of resting breathing at normoxia (on left) and 10% hypoxia (on right) obtained in one rat per experimental group: **(A)** and **(B)** Sham (in green) at W0 and W1, respectively; **(C)** and **(D)** low-dose bleo (in purple) at W0 and W1, respectively; **(E)** and **(F)** high-dose bleo (in blue) at W0 and W1, respectively.

**FIGURE 3:**
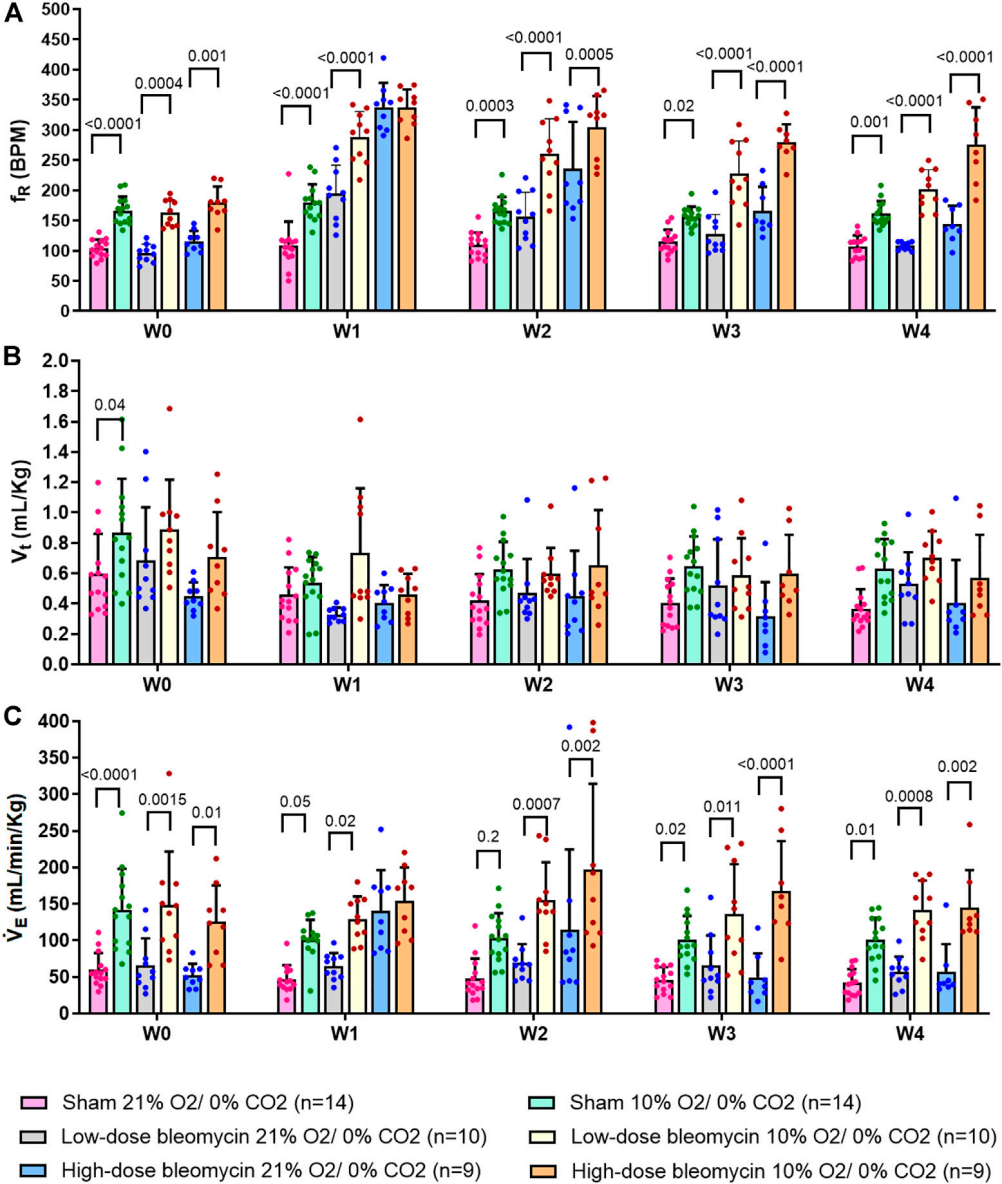
Effect of saline and (low-dose and high-dose) bleomycin on ventilatory parameters in sham (*n* = 14), low-dose bleo (*n* = 10) and high-dose bleo (*n* = 9) rats. Two-way ANOVA, Values are mean ± SD: **(A)** Respiratory rate (f_R_) at normoxia vs. 10%; **(B)** Tidal volume (V_t_) at normoxia vs. 10% hypoxia; **(C)** Minute ventilation (V_E_) at normoxia vs. 10% hypoxia.

**FIGURE 4:**
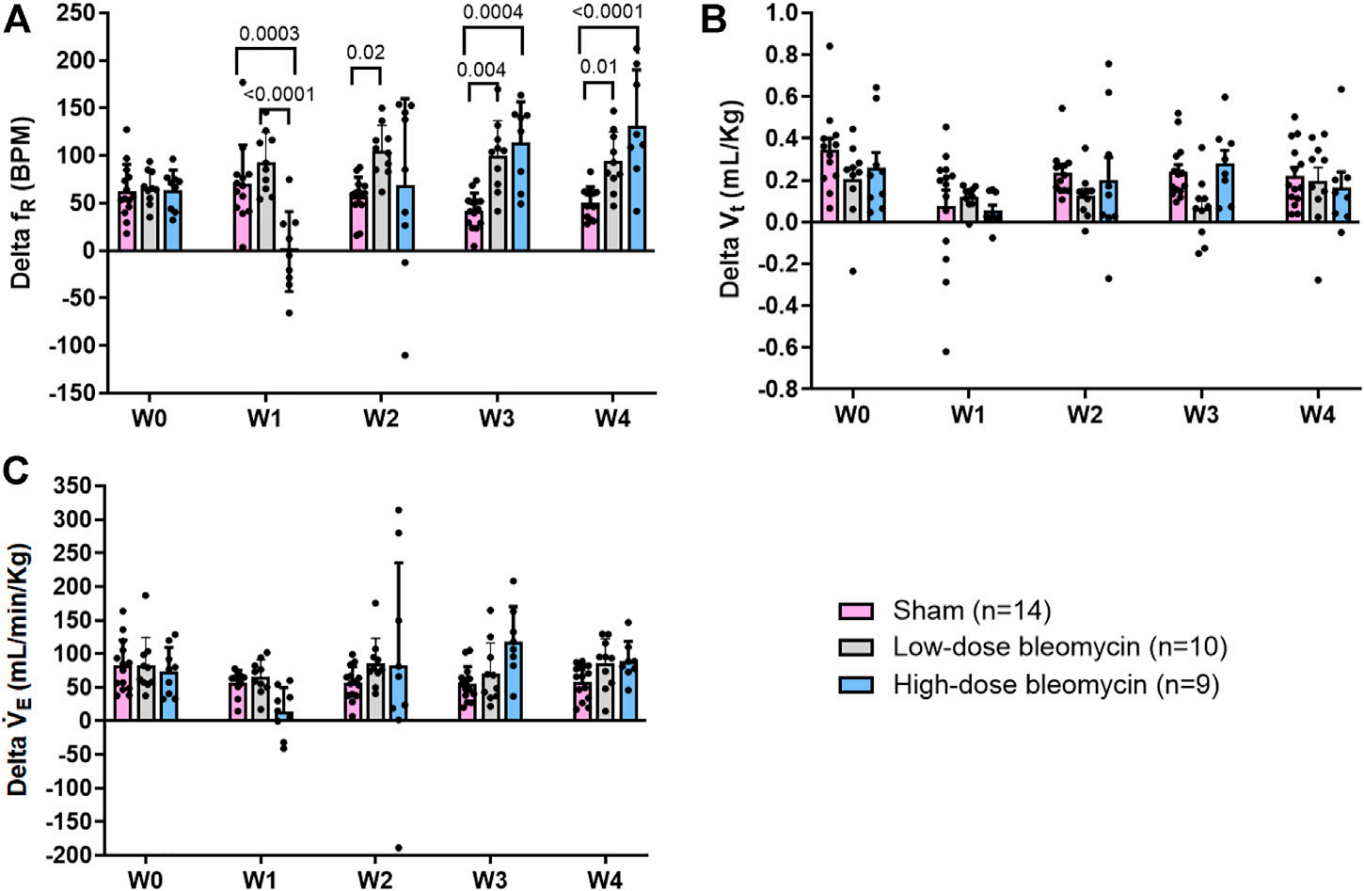
**(A)** Delta f_R_; **(B)** Delta V_t_; **(C)** Delta V_E_ in response to 10% hypoxia in sham (*n* = 14), low-dose bleo (*n* = 10) and high-dose bleo (*n* = 9) rats.

As noted in Figures 2, 3A and Table 2 Sham rats showed a consistent normal resting f_R_ (180 ± 12 bpm) at W1 post-saline administration compared to W0 (104 ± 15 bpm). On the other hand, resting f_R_ increased (*p* < 0.0001) in both low-dose bleo- (193 ± 55 bpm) and high-dose bleo-treated rats (333 ± 46 bpm) at W1 post-bleo administration as compared to their baseline at W0 (low-dose group = 97 ± 16 bpm and high-dose group = 117 ± 21 bpm). The resting f_R_ in low- and high-dose bleo rats was partially restored by W3 (127 ± 32 bpm and 166 ± 40 bpm, respectively) and W4 (108 ± 7 bpm and 144 ± 30 bpm, respectively). These changes in resting f_R_ also influenced similar trends in resting V_E_ (Figures 3C, 4C). No significant changes were seen for the change in V_t_ in either group (Figures 3B and 4B).

**TABLE 2:** Mean resting respiratory rate (f_R_) (in BPM) for sham, low-dose bleo and high-dose bleo rats.

Mean resting f_R_ (BPM)					
	W0	W1	W2	W3	W4
Sham (*n* = 14)	108 ± 12	104 ± 15	107 ± 26	118 ± 25	99 ± 16
Low-dose bleo (*n* = 10)	97 ± 16	193 ± 55###	151 ± 41##	125 ± 31	103 ± 12
High dose-bleo (*n* = 9)	117 ± 21	333 ± 46ǂǂǂǂ	238 ± 81ǂǂ	198 ± 48ǂǂǂ	164 ± 47ǂǂ

Values are mean ± SD; One-way ANOVA with bonferroni multiple comparison test; f_R_ indicates respiratory rate; bleo indicates bleomycin. ##*p* = 0.007 (Low-dose bleo- W0 vs. W2), ###*p* = 0.001 (Low-dose bleo- W0 vs. W1), ǂǂ*p* = 0.007 (High-dose bleo- W0 vs. W2), ǂǂ*p* = 0.01 (High-dose bleo- W0 vs. W4), ǂǂǂ*p* = 0.001 (High-dose bleo- W0 vs. W3), ǂǂǂǂ*p* = 0.0001 (High-dose bleo- W0 vs. W1)

### Effects of hypoxia on respiration

The peripheral chemoreflex was activated by challenging the rats with 10% hypoxia for a duration of 5 min and ventilatory parameters were assessed. The chemoreflex was assessed by measuring the absolute difference between 21% O_2_/0% CO_2_ and 10% O_2_/0% CO_2_. At baseline (W0), f_R_ increased in response to 10% O_2_ in sham, low-dose and high-dose bleo-treated rats by 63 ± 29 bpm, 66 ± 18 bpm and 64 ± 21 bpm, respectively, before saline and bleo administration on day 1 (Figures 2A, 4A). 1W- post-saline/bleo administration, sham rats continued to respond normally to 10% hypoxic gas challenge. While the low-dose bleo-treated rats exhibited an activation of the chemoreflex with a significant increase in f_R_ (∆f_R_ = 93 ± 31 bpm, *p* < 0.0001). The high-dose bleo-treated rats, on the other hand, exhibited a significant blunting of the chemoreflex response (∆f_R_ = −0.97 ± 42 bpm, *p* < 0.0001) compared to sham rats with ∆ f_R_ of 70 ± 41 bpm at 1W- post-bleo administration (Figures 2A, 4A).

For the high-dose bleo-treated rats, changes in V_E_ (∆V_E_) were not statistically significant (*p* = 0.8). However, we see a pattern of blunted ∆V_E_ at W1 (∆V_E_ = 57 ± 18 ml/min/Kg and 14 ± 35 ml/min/Kg in sham and high-dose bleo-treated rats, respectively) due to a blunted ∆f_R_ without significant changes in ∆V_t_ (Figure 4C). Sham rats continued to show a normal and consistent chemoreflex activation at W2 (∆f_R_ = 42 ± 19 bpm), W3 (∆f_R_ = 41 ± 19 bpm) and W4 (∆f_R_ = 50 ± 17 bpm). Low-dose bleo-treated rats exhibited an increased chemoreflex activation at W2 (∆f_R_ = 105 ± 26 bpm, *p* = 0.02) and a significant sensitization at W3 (∆f_R_ = 100 ± 37 bpm, *p* = 0.03) and at W4 (∆f_R_ = 94 ± 31 bpm, *p* = 0.006) when compared to the sham group for respective time points (Figure 4A). High-dose bleo-treated rats showed a restoration in chemoreflex activation at W2 (∆f_R_ = 69 ± 91 bpm) and show a chemoreflex sensitization at W3 (∆f_R_ = 114 ± 43 bpm, *p* = 0.007) and W4 (∆f_R_ = 132 ± 58 bpm, *p* = 0.001) when compared to the sham group for respective time points (Figure 4A). Similar trends were observed for ∆V_E_ in all experimental groups (Figure 4C). It is important to note that irrespective of the dose of bleomycin, there was sensitization of chemoreflex around W3 and 4. V_t_ was not significantly changed in either group (Figure 4B).

### Effects of normoxic-hypercapnia on respiration

**FIGURE 5:**
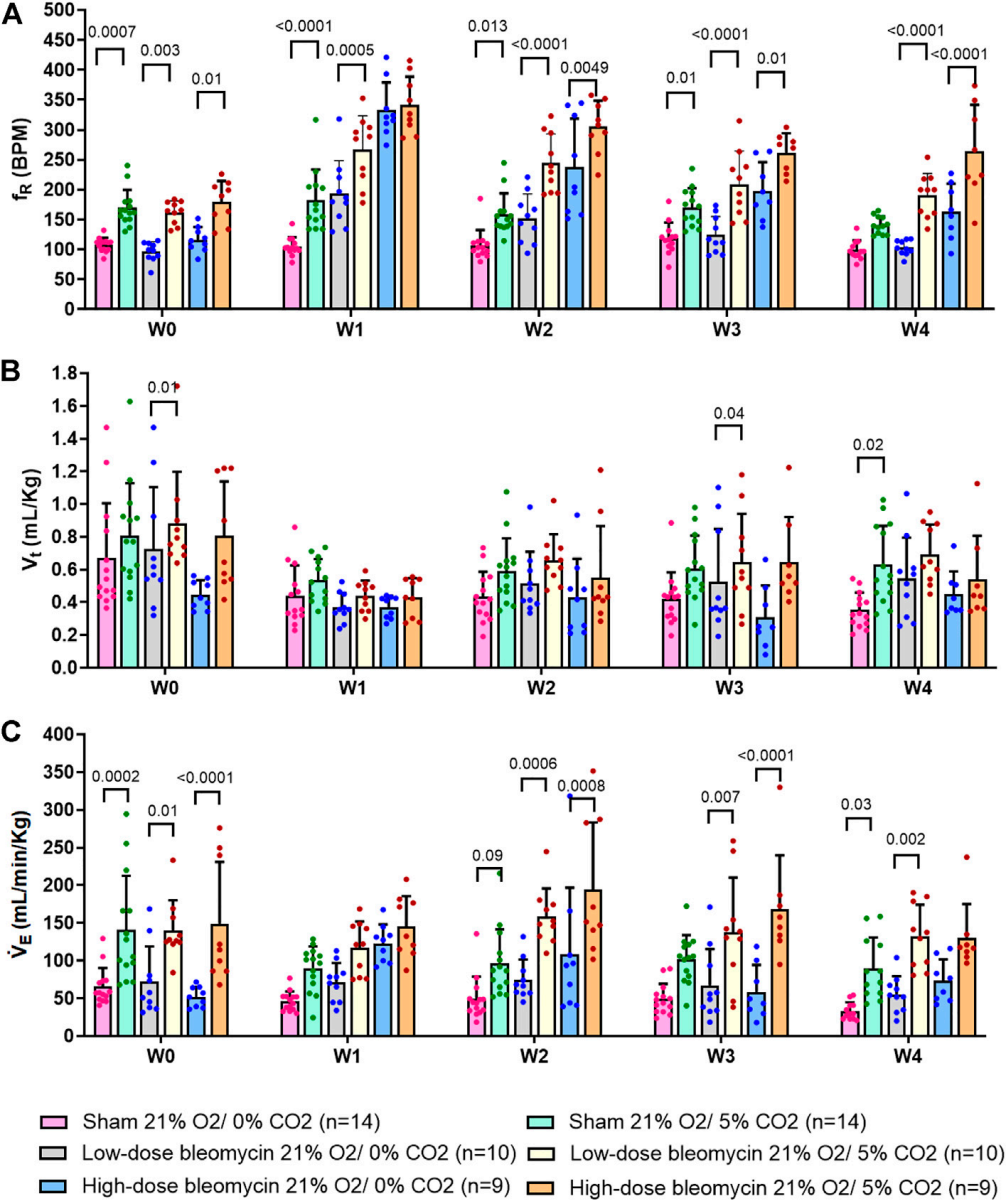
Effect of saline and (low-dose and high-dose) bleomycin on ventilatory parameters in sham (*n* = 14), low-dose bleo (*n* = 10) and high-dose bleo (*n* = 9) rats. Two-way ANOVA, Values are mean ± SD: **(A)** Respiratory rate (f_R_) at normoxia vs. normoxic-hypercapnia; **(B)** Tidal volume (V_t_) at normoxia and normoxic-hypercapnia; **(C)** Minute ventilation (V_E_) at normoxia vs. normoxic-hypercapnia.

**FIGURE 6:**
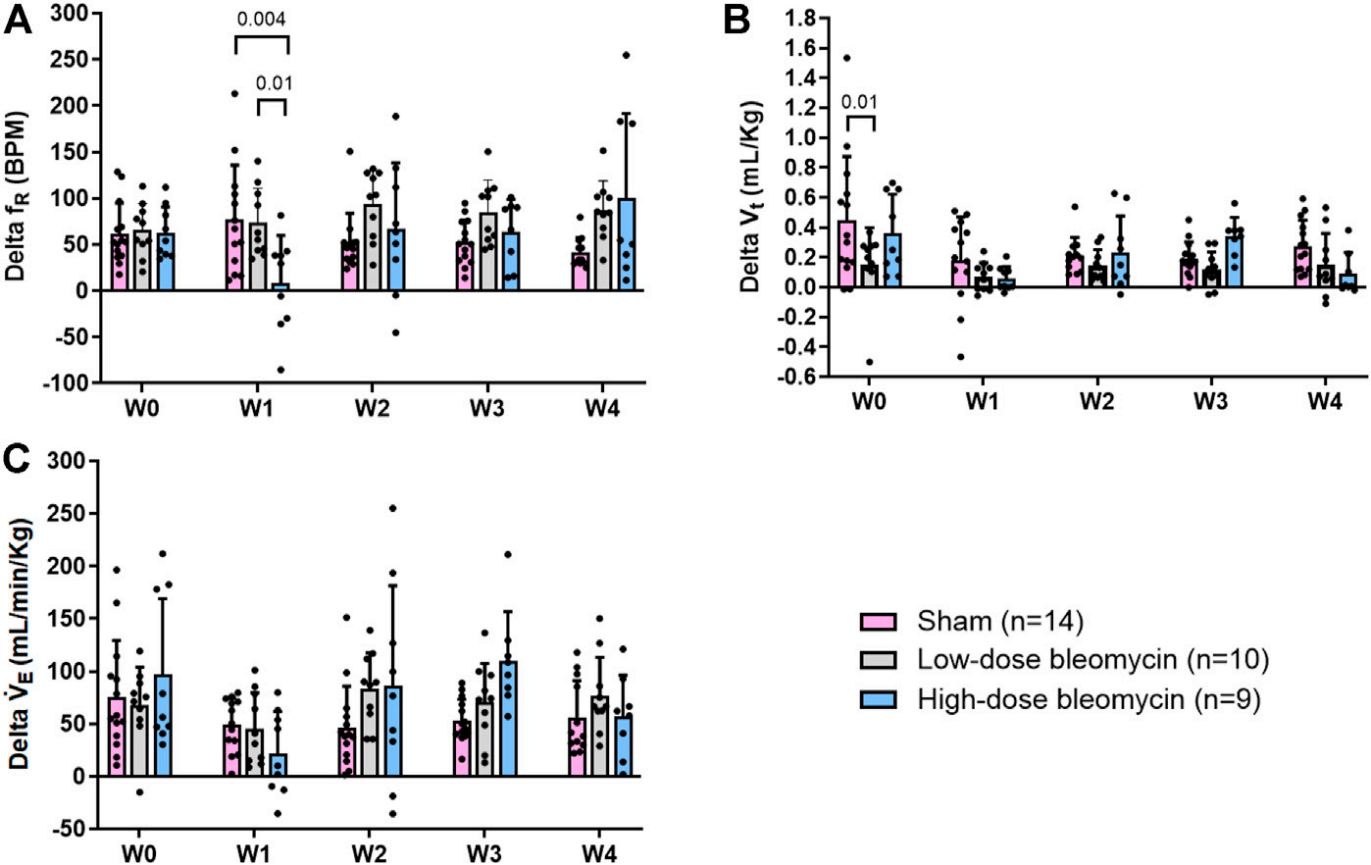
**(A)** Delta f_R_; **(B)** Delta V_t_; **(C)** Delta V_E_ in response to normoxic-hypercapnia in sham (*n* = 14), low-dose bleo (*n* = 10) and high-dose bleo (*n* = 9) rats.

In the same groups of rats, both peripheral and central chemoreflexes were activated by challenging the rats with 5% CO_2_/21% O_2_ for a duration of 5 min. The chemoreflex was assessed by measuring the absolute difference between 21% O_2_/0% CO_2_ and 21% O_2_/5% CO_2_. At baseline (W0), f_R_ was increased in both sham and bleo-treated rats (low-dose and high-dose) by 62 ± 33 bpm, 66 ± 28 bpm and 63 ± 28 bpm before saline or bleo administration, respectively (Figures 5A, 6A). At W1 post-saline/bleo administration, sham rats continued to respond normally to 5% hypercapnic/21% normoxic gas challenge (∆f_R_ = 77 ± 59 bpm). The low-dose bleo-treated rats exhibit a chemoreflex response to normoxic-hypercapnia (∆f_R_ = 74 ± 37 bpm) while the high-dose bleo-treated group showed a blunted chemoreflex response by a significant reduction in ∆f_R_ to 8 ± 51 bpm (*p* = 0.004) compared to its timed-control (sham rats at W1) (Figure 6A). This blunted chemoreflex response was similar to the blunted peripheral chemoreflex response 1W post-bleo instillation.

Changes in ∆V_E_ showed a similar blunted pattern in bleo-treated rats at W1 (∆V_E_ = 49 ± 25 ml/min/Kg, 46 ± 34 ml/min/Kg and 22 ± 40 ml/min/Kg in sham, low-dose, and high-dose bleo-treated rats, respectively) (Figures 5C and 6C) due to increased f_R_ without significant changes in V_t_ (Figures 5B and 6B) but were not statistically significant. Sham rats continued to show normal chemoreflex activation at W2 (∆f_R_ = 52 ± 32 bpm), W3 (53 ± 24 bpm) and W4 (42 ± 16 bpm). Although not statistically significant, low-dose bleo-treated rats showed a pattern of sensitized chemoreflex at W2 (93 ± 36 bpm), W3 (85 ± 35 bpm) and W4 (100 ± 92 bpm) just as seen for the peripheral chemoreflex in response to 10% hypoxia (Figure 6A). High-dose bleo-treated rats showed a restoration in chemoreflex activation at W2 (67 ± 71 bpm) and 3 (64 ± 35 bpm) and was sensitized at W4 (100 ± 92 bpm, *p* = 0.01) (Figure 6B). Similar trends were observed for ∆V_E_ in both experimental groups throughout the experimental timeline (Figure 6C).

### Effects of hyperoxia on respiration 1-week post-bleo instillation

**FIGURE 7:**
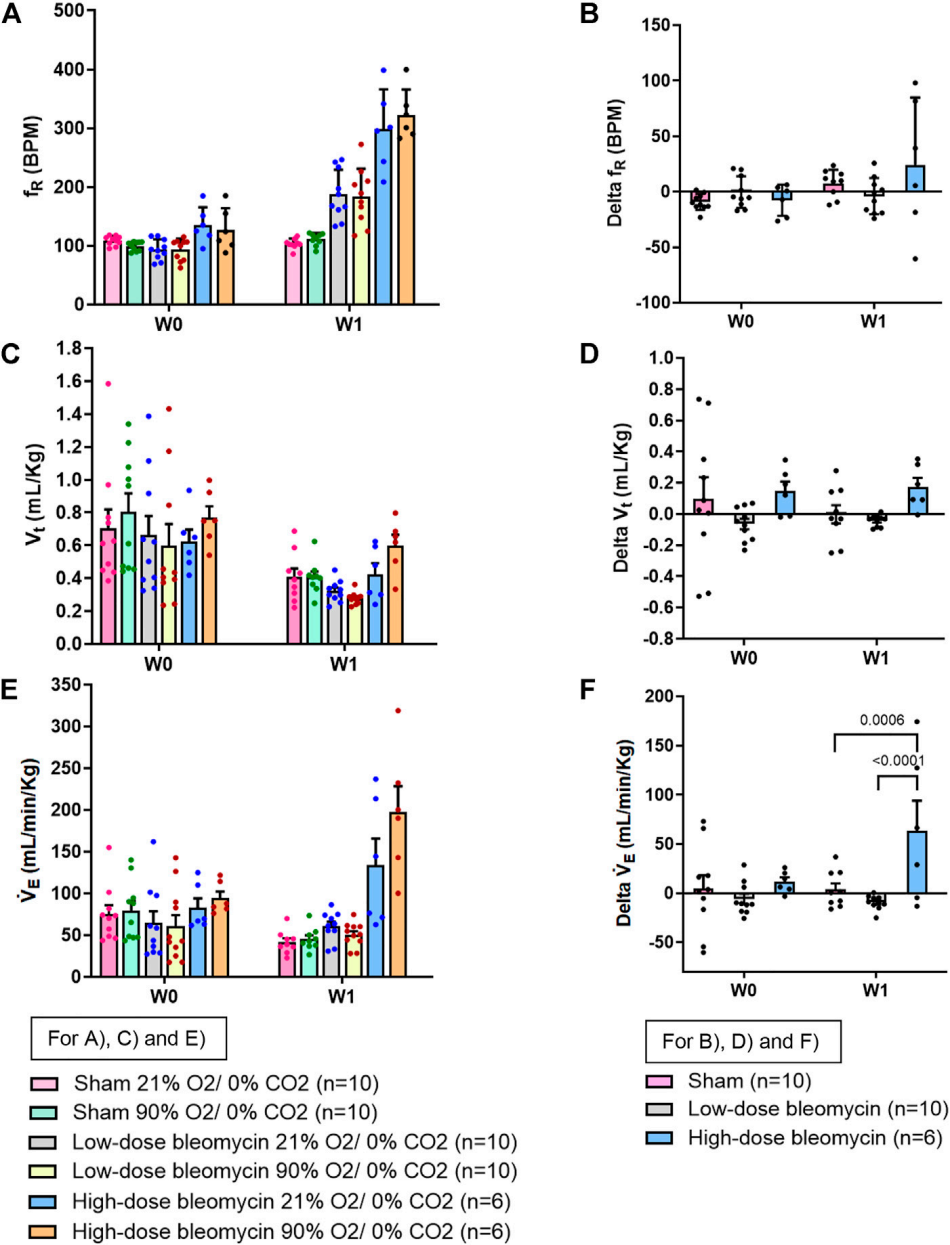
Effect of 90% hyperoxia on ventilatory parameters in sham (*n* = 10), low-dose bleo (*n* = 10) and high-dose bleo (*n* = 6) rats at W0 and W1. Two-way ANOVA, Values are mean ± SD: **(A)** Respiratory rate (f_R_) at normoxia vs. 90% hyperoxia; **(B)** Delta f_R_; **(C)** Tidal volume (V_t_) at normoxia vs. 90% hyperoxia; **(D)** Delta V_t_; **(E)** Minute ventilation (V_E_) at normoxia and 90% hyperoxia; **(F)** Delta V_E_.

To inhibit tonic chemoreflex activation at rest, sham and bleo rats were exposed to 90% O_2_/0% CO_2_ gas mixture at W1 and ventilatory parameters were evaluated. No significant change in f_R_ was seen. (Figure 7A). 1W post-saline/bleo instillation (low- and high-dose), ∆f_R_ for sham vs. low dose and high-dose bleo-treated rats was 8 ± 12 bpm, −4 ± 16 bpm and −6 ± 17 bpm, respectively (Figure 7B). No significant changes were seen for V_t_ in either group (Figures 7C,D). V_E_ was significantly higher in high-dose bleo group when compared to sham and low-dose bleo group (Figures 7E,F).

## Discussion

The major findings of the present study are as follows: 1) both low-dose and high-dose bleo-treated rats showed a significant increase in resting f_R_ W1 post-bleo administration which was partially restored to normal by W3 and 4; 2) In response to 10% hypoxia and 5% normoxic-hypercapnia, while low-dose bleo-treated rats exhibited an activated peripheral chemoreflex response, the high-dose bleo-treated rats showed a blunted chemoreflex response at W1 post-bleo administration; 3) At W3 and 4, post-recovery from ALI, in response to 10% hypoxia and 5% normoxic-hypercapnia, both low-dose and high-dose bleo-treated rats exhibited a sensitized chemoreflex response; 4) In response to hyperoxia (90% O_2_), both low-dose and high-dose treated bleo rats showed no significant reduction in the increased f_R_ at W1 post-bleo administration.

Shortness of breath and an increase in resting f_R_ caused by impairment of gas exchange are the main characteristics of ALI/ARDS which accounts for 10% of ICU admissions and has a high mortality rate (Mowery et al., 2020). Impaired gas exchange in ALI stimulates chemoreflex activation (Jacono et al., 2006). A time-course study of changes in chemoreflex activation during ALI has never been explored. The present study assesses the functional changes in chemoreflex activation over a 4-week time-course fashion from the beginning of ALI and during its recovery.

There are multiple ALI animal models reported in the scientific literature (Matute-Bello et al., 2008). However, no single model is the “best” model for ALI. We utilized the bleo model, which is widely used to model ALI (Moore and Hogaboam, 2008). Intra-tracheal administration of bleo damages the alveolar endothelium (Matute-Bello et al., 2008). Two different doses of bleo were used with the aim to study differences in the severity of ALI. Other common ALI animal models include the LPS model, which is also very reproducible and is widely used to study ALI (Zeng et al., 2017; Ye and Liu, 2020; Hou et al., 2021). Unfortunately, for the purpose of our current study, it could not be used because LPS is known to directly affect the sympathetic and parasympathetic ganglia (Shadiak et al., 1994; Hosoi et al., 2005; Kunda et al., 2014; Blum et al., 2017; Boucher et al., 2018) and for this reason, we think that LPS alone could directly interfere with the function of the carotid bodies thus confounding the primary goal of examining the effect of ALI on the chemoreflex.

Poor gas exchange caused by disruption of the normal alveolar-capillary endothelial barrier in this disease condition leads to systemic hypoxemia that is known to activate the peripheral chemoreceptors, which is one of the primary defense mechanisms to restore normal gas levels in the body (Dias-Freitas et al., 2016). Although there are some studies that provide possible mechanistic explanations but the neural mechanism(s) that drive the increase in f_R_ are not fully understood. The existing literature provides evidence that the chemoreflex function is altered during ALI (Jacono et al., 2006; Huxtable et al., 2011). A study by Jacono et al., 2006 showed that the chemoreflex, 5 days post-ALI, was sensitized in bleo-treated rats. This is consistent with what we see in our W1 post-low-dose bleo treated rats. Another study by Huxtable et al., 2012 used an LPS-ALI rat model to show an increase in resting f_R_ and a blunted chemoreflex response to 10.5% O_2_, 7%CO_2_ gas challenge at day 1 post-LPS (i.p.) treatment. Our study showed that at W1 post-bleo treatment, while the low-dose bleo-treated rats exhibited a pattern of sensitized chemoreflex activity, the high-dose bleo-treated rats on the other hand exhibited a blunted chemoreflex response to 10% hypoxia. We initially thought that this may be possible due to a “ceiling effect”, meaning that the chemoreflex could be maximally activated at baseline breathing normal (21% O_2_) air. This possibility was ruled out by exposing the bleo-rats to hyperoxia with the goal to inhibit the chemoreflex (Figures 7A,B). Interestingly, we did not see a change in f_R_ in that group of rats. It is important to note that in this study, we are testing the chemoreflex activation in response to hypoxia, and both peripheral and central chemoreflex activation in response to normoxic-hypercapnia as a stimulus. We know that the CBs also get activated by changes in pH level which can act as a potential stimulus for chemoreflex activation in ALI condition. We have previously provided evidence that compared to vehicle-treated rats, bleo rats exhibit significantly decreased blood pO_2_ and sO_2_ at W1 post-bleo, indicating severe hypoxemia during acute lung injury. Bleo rats also exhibit elevated pCO_2_ and decreased pH at W1 post-bleo, indicating blood acidosis (Kitzerow et al., 2022). Therefore, the role of other stimuli (such as, pH) in tonic activation of chemoreceptors during severe ALI condition require further investigation.

During the recovery from ALI, there was a partial restoration of the resting fR in bleo rats at W3 and W4. Histological proof of recovery from ALI post-intratracheal delivery of bleo has shown by many groups over the years (Moore and Hogaboam, 2008; Young et al., 2019; Mahmutovic Persson et al., 2020). Interestingly, at W3 and W4 post-ALI, the chemoreflex was sensitized in response to hypoxia and normoxic-hypercapnia in both low-dose- and high-dose bleo groups. We show for the first time to our knowledge, that chemoreflex activity during recovery from ALI is sensitized. The present study did not explore what causes the chemoreflex to be sensitized during recovery from ALI. It is possible that this may be attributed to multiple effects mediated at one or more levels of the reflex arc. Although the recovery phase of the ALI has not been extensively studied in terms of mechanisms for sensitized chemoreflex activity, it is already known that ALI influences both the pattern and the rhythm of breathing. In early stages of ALI (W1 post-bleo), Jacono et al. detected increased immunoreactivity of the pro-inflammatory cytokine interleukin-1β in the NTS from ALI rats. Bilateral micro-injections of IL-1β in the NTS of naïve rats showed increased f_R_ similar to that found in rats with ALI. They speculated that though initiated by sensory input, the characteristic ventilatory pattern after lung injury may be mediated by neuro-inflammation by cytokines in the NTS (Getsy et al., 2019; Hsieh et al., 2020; Litvin et al., 2020). Based on their evidence, it would not be surprising to expect similar plasticity in the NTS during the recovery stages of ALI. Our lab previously provided evidence of neuroinflammation and altered (increased) neuronal excitability in the stellate ganglia during the recovery phase of bleo-induced ALI (Hong et al., 2021). Based on this and our data for sensitized chemoreflex response to hypoxic stimulus, it is reasonable to speculate that similar neuroinflammation is possible in the carotid bodies which could lead to an altered chemoreflex function in this disease condition. In addition, a similar mechanism could also be possible in the superior cervical ganglion (SCG) that is located on the same sympathetic chain as the stellate ganglia. The post ganglionic axons of the SCG are known to innervate the CBs (Iturriaga et al., 2016). Electrical stimulation of the SCG has been shown to significantly sensitize the CB chemoreflex in hypertensive and normotensive rats (Felippe and Paton, 2021; Getsy et al., 2021; Felippe et al., 2022). Therefore, an increased SCG neuronal activity during the recovery of ALI, in-part, might also contribute to chemoreflex sensitization observed in our study. In addition, systemic hypoxia is a major consequence in chronic heart failure (CHF) condition (Morrissey et al., 2011) and studies provide evidence that experimental models of CHF (Andrade et al., 2015) and CHF patients (Giannoni et al., 2009) exhibit sensitized chemoreflex that contributes to sympatho-excitation and disordered breathing (Morrissey et al., 2011). Like CHF, ALI also leads to systemic hypoxia and a sensitized chemoreflex function in response to hypoxia. In addition, the increase in Ang II-dependent oxidative stress has been shown to contribute to altered CB function in CHF (Andrade et al., 2015). Interestingly, studies show that ALI results in decreased ACE2 expression and increased production of Ang II in the acid aspiration mice model of ALI (Imai et al., 2005). Therefore, Ang II signaling could be a potential mechanism causing sensitized chemoreflex during the recovery phase of ALI.

## Conclusion

In summary, this study provides evidence that in the early phase of ALI, the chemoreflex is sensitized during low-dose ALI (moderate ALI) and blunted during high-dose bleo ALI (severe ALI). More importantly, it brings attention to the novel discovery of a sensitized chemoreflex during recovery from both moderate and severe ALI. The chemoreflexes are important modulators of sympathetic activation. It is well established that acute and/or chronic activation of the –chemoreflex enhances sympathetic drive (Plataki et al., 2013). Excessive sympathetic outflow can lead to cardiac arrythmias, cardio-renal syndrome, metabolic syndrome, T2 diabetes and deterioration of cardiac function (Paton et al., 2013; Conde et al., 2014; Iturriaga et al., 2016; Del Rio et al., 2017; Cunha-Guimaraes et al., 2020). Therefore, identification and further understanding of the neural mechanism that mediates changes in chemoreflex function during early and late stages of ALI will provide important information for the long-term goal of development of novel targeted therapeutic approaches to improve clinical outcomes.

## Data Availability

The original contributions presented in the study are included in the article/Supplementary Material, further inquiries can be directed to the corresponding author.
